# VIRGO: visualization of A-to-I RNA editing sites in genomic sequences

**DOI:** 10.1186/1471-2105-14-S7-S5

**Published:** 2013-04-22

**Authors:** Rosario Distefano, Giovanni Nigita, Valentina Macca, Alessandro Laganà, Rosalba Giugno, Alfredo Pulvirenti, Alfredo Ferro

**Affiliations:** 1 Department of Mathematics and Computer Science - University of Catania, Catania, Italy; 2 Department of Molecular Virology, Immunology and Medical Genetics Comprehensive Cancer Center - The Ohio State University, Ohio, USA; 3 Department of Clinical and Molecular Biomedicine - University of Catania, Catania, Italy

## Abstract

**Background:**

RNA Editing is a type of post-transcriptional modification that takes place in the eukaryotes. It alters the sequence of primary RNA transcripts by deleting, inserting or modifying residues. Several forms of RNA editing have been discovered including A-to-I, C-to-U, U-to-C and G-to-A. In recent years, the application of global approaches to the study of A-to-I editing, including high throughput sequencing, has led to important advances. However, in spite of enormous efforts, the real biological mechanism underlying this phenomenon remains unknown.

**Description:**

In this work, we present VIRGO (http://atlas.dmi.unict.it/virgo/), a web-based tool that maps Ato-G mismatches between genomic and EST sequences as candidate A-to-I editing sites. VIRGO is built on top of a knowledge-base integrating information of genes from UCSC, EST of NCBI, SNPs, DARNED, and Next Generations Sequencing data. The tool is equipped with a user-friendly interface allowing users to analyze genomic sequences in order to identify candidate A-to-I editing sites.

**Conclusions:**

VIRGO is a powerful tool allowing a systematic identification of putative A-to-I editing sites in genomic sequences. The integration of NGS data allows the computation of p-values and adjusted p-values to measure the mapped editing sites confidence. The whole knowledge base is available for download and will be continuously updated as new NGS data becomes available.

## Background

RNA Editing is a type of post-transcriptional modification that takes place in eukaryotes. It alters the sequence of primary RNA transcripts by deleting, inserting or modifying residues. Several forms of RNA editing have been discovered including A-to-I, C-to-U, U-to-C and G-to-A. Here we focus on A-to-I editing (Adenosine-to-Inosine), the most frequent and common one [1]. Adenosine (A) deamination produces its conversion into inosine (I), which, in turn, is interpreted by both the translation machinery and the splicing machinery [2] as guanosine (G). Since inosine binds cytosine (C), the A-U base pairs in the secondary structure are changed into I:U mismatches [3]. This biological phenomenon is catalyzed by enzymes members of the Adenosine Deaminase Acting on RNA (ADAR) family and occurs only on dsRNA structures [1,4,5].

The A-to-I RNA editing may be either *promiscuous *or *specific*. The *promiscuous *RNA editing occurs within long duplexes [6,7], while *specific *RNA editing A-to-I occurs within shorter duplex regions, often formed by an exon and an intron sequence [8]. Moreover, it has been reported that A-to-I RNA editing can target both exonic and intronic regions as well as 5' and 3'-UTRs regions. This can have different consequences in the biogenesis of mRNA [2,9], the translation [1], the mRNA export from the nucleus to the cytoplasm [10,11], and the degradation of I-containing mRNA molecules [12]. In the last few years, it has been reported that RNA editing may occur in small noncoding RNA molecules in particular within precursor-tRNA [13] and pri-miRNAs [14,15]. It has been estimated that ~ 16% of these sequences undergo A-to-I editing [14], influencing the pri-miRNA's maturation process [16] and, consequently, the recognition of binding sites on target mRNAs [17-19].

It is well known that the activity of RNA editing is higher in mRNAs of mammalian brain than other tissues [20] and this leads to the assumption that editing plays a crucial role in the central nervous system [3]. Therefore, malfunctions of ADARs could lead to serious consequences, in particular it has been observed that an imbalance of ADAR expression/activity induces a variety of human diseases [21].

A common approach to identify putative A-to-I editing sites relies on the alignment of the cloned cDNA gene sequence to its genomic sequence highlighting A-to-G mismatches. Recent literature reports different screenings designed to detect A-to-I RNA editing sites in human, especially in ALU-type repetitive elements located also in UTRs regions [6,7,22-25]. Li et al. [26] presented an unbiased assay to select more than 36, 000 computationally predicted non-repetitive A-to-I sites. The sites were detected using amplified and sequenced padlock probes. The authors used cDNA and gDNA from several tissues and derived from a single individual. These methods led to the discovery of thousands of ADAR substrates which may help clarify the function of A-to-I RNA editing on the regulation of gene expression and quantify the impact of A-to-I editing on transcriptome and proteome diversity. Eggington et al. [27] provide a web-based application which predicts editing sites in dsRNA of any sequence using Sanger sequencing protocols to perform a more accurate quantitative analysis. More recently, in contrast to the previous approaches, new methods, based on Next Generation Sequencing (NGS) data, have been developed to identify A-to-I editing sites [28-32]. These new approaches have allowed the detection of novel editing sites within coding and non-coding genes [33]. On the other hand they produced a high number of false editing sites, since the NGS technology is prone to error [31].

Few systems are available on the web. *dbRES *[34] was the first web-oriented database for annotated RNA editing sites, but the last update goes back to 2007 and contains only a few dozen of human editing sites. More recently, Kiran and Baranov created DARNED [35], the largest database of human RNA editing sites providing a centralized access to published data. RNA editing locations are mapped on the reference human genome. DARNED is periodically updated and contains more than 300,000 editing sites, but no statistical significance is provided. In 2011, Picardi et al. presented Expedit [29]. It is a web application that maps data and, given individual sequence reads as input, executes a comparative analysis against DARNED editing sites. No statistical significance of results is given.

In this work, we present VIRGO (Visualization of A-to-I RNA editing sites into GenOmic sequences, http://atlas.dmi.unict.it/virgo/), a knowledge-base equipped with a web-interface allowing users to map putative and known A-to-I editing sites into gene regions (including coding sequences, introns, and UTRs). We consider as putative editing sites A-to-G mismatches between genomic and EST sequences, while known A-to-I editing sites are obtained from DARNED.

**Figure 1:**
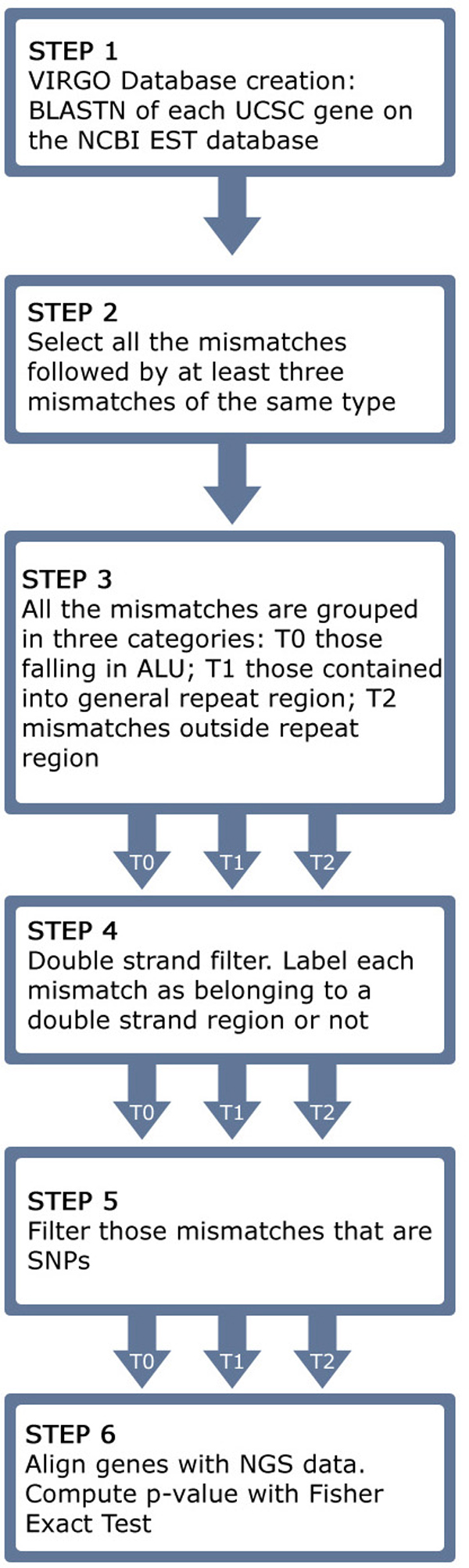
**Sequence of steps to identify putative A-to-I editing sites**. The pipeline that VIRGO uses to built the knowledge base.

VIRGO borrows from literature the basic computational techniques that are used to identify A-to-G mismatches as putative editing sites. These bioinformatics methods and resources (i.e. alignment between genomic and EST sequences, clustering, double strand RNA region identification, Next Generation Sequencing data) are then integrated into a workflow (see Figure 1) allowing users to facilitate the analysis of genomic sequences.

In particular, the VIRGO knowledge-base has been created by matching all the human genes regions obtained from UCSC (hg19) to the EST database using filters and NGS data. The filters allow the selection of candidate editing events in clusters [36], lying in repeated and double strand regions and not classified as SNPs. Moreover, VIRGO locally maps all the editing events stored in DARNED. This feature allows the visualization of all DARNED editing sites through the VIRGO web interface. Finally, VIRGO uses the DARNED editing sites for which NGS information is available to compute the expected frequencies of A to G substitution that can happen in a mismatch aligned column. This knowledge is then used to compute p-values for all VIRGO editing events for which NGS information is available.

The VIRGO web interface allows annotation of genomic sequences, provided by users, known editing sites and those sites passing the filters described above.

### Construction and content

VIRGO is a knowledge base that integrates information retrieved from specialized biological databases. The core of the system has been developed in C++, while the front-end consists of a web interface developed in PHP.

The data integration process implemented in VIRGO consists of a sequence of steps carried out to identify putative A-to-I editing sites (see Figure 1). The database construction, which has been done offline, includes six steps. All filters are mandatory, therefore, a site that does not pass one of such steps is discarded. The last step is applied only when mismatches align with the NGS reads.

The steps are described below.

**Step 1**. We downloaded the whole set of human genes from UCSC (http://genome.ucsc.edu/buildGRCCh37/hg19). Then, using BLASTN, we aligned all the genes with NCBI EST database. Although this step is very time consuming, it allows us to identify all the potential A-to-I editing sites. VIRGO creates an initial database by selecting the A-G mismatches between the genes and the EST sequences.

**Figure 2:**
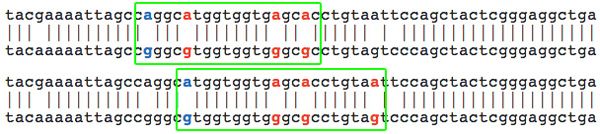
**Clustering filter**. The A-G mismatch in blue color is followed by three mismatches of the same type (in red color). Furthermore, no gaps are present. The three mismatches following the initial candidate editing site are included as putative editing events and are highlighted in the alignment with ESTs.

**Step 2**. According to [36], editing events usually happen in cluster. After binding the mRNA, ADAR creates bunches of close editing events. An edited sequence typically shows editing in many close-by sites. Therefore, it is very unlikely to observe isolated editing events inside a sequence. *The clustering filter *implements the methodology presented in [36] by selecting A-G mismatches that are followed by at least three mismatches of the same kind, without gaps or other types of mismatches (see Figure 2 for an example).

**Step 3**. VIRGO partitions the selected mismatches in three categories. To achieve that, we label the genes as falling in ALU regions (T0), in repeat regions (T1), and in non repeat region (T2).

**Step 4**. VIRGO verifies whether mismatches (from all the classes created above) occur into double-stranded regions. For this purpose we applied a technique already used in [6,36] for the prediction of the double strand portion of a RNA secondary structure. It creates a short reverse complementary sequence centered on each mismatch by retrieving upstream and downstream flanking nucleotides. Then it searches for the constructed reverse complementary sequence into the gene where the mismatch has been found. In particular, when a mismatch occurs into an ALU repetitive region the length of the short complementary sequence is equal to the length of the ALU region. Otherwise, the length of the short sequence is equal to 251 nucleotides including the mismatch.

**Figure 3:**
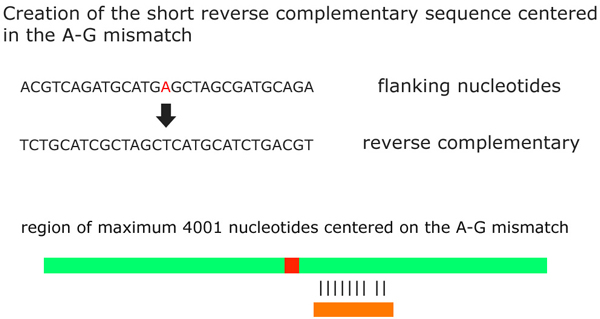
**Fourth Step**. VIRGO verifies whether mismatches occur into double-stranded regions by creating a short reverse complementary sequences centered on the mismatch. VIRGO aligns the created sequence with a region of maximum 4001 nucleotides centered on the A-G mismatch. If the percentage of the alignment is greater than or equal to 85%, VIRGO annotates that mismatch as occurring into a double-strand region.

Next, VIRGO aligns the created sequence with a region with no more than 4001 nucleotides centered on the A-G mismatch. Since the length of the reverse complement in ALU and repeat regions is not constant we set the minimum length for the alignments to be 85% of the length of the sequence (i.e. the alignment consists of at least 214 nucleotides over 251). Consequently, in the alignment we look for an identity of at least 85%. VIRGO annotates that mismatch as occurring into a double-strand region [6,36] (see Figure 3 for an example).

**Step 5**. VIRGO, uses the database *All SNPs(135) *contained in UCSC, to filter the mismatches that are already classified as SNPs.

**Step 6**. VIRGO performs an alignment of the genes with a subset of NGS data taken from the following experiments: **SRP002274 - GSE19166 **(http://www.ncbi.nlm.nih.gov/Traces/sra/sra.cgi?study=SRP002274) and **SRP007465 **(http://www.ncbi.nlm.nih.gov/Traces/sra/sra.cgi?study=SRP007465).

The subset of short reads is constructed as follows. Alignment of human genome with short reads is performed by *BOWTIE *[37]. In order to reduce noise, only the best alignments with at most two mismatches by using -*a *and -*v *parameters are accepted. By specifying -*a*, VIRGO instructs *BOWTIE *to report all valid alignments, subjected to the alignment policy -*v 2 *(at most two mismatches are allowed).

The selected short reads are mapped on each VIRGO mismatch, selecting those mismatches occurring into at least five short reads. This alignment allows to compute, for some of the editing events, p-value and adjusted p-value yielding the confidence that the candidate mismatch is not a false positive.

**Figure 4:**
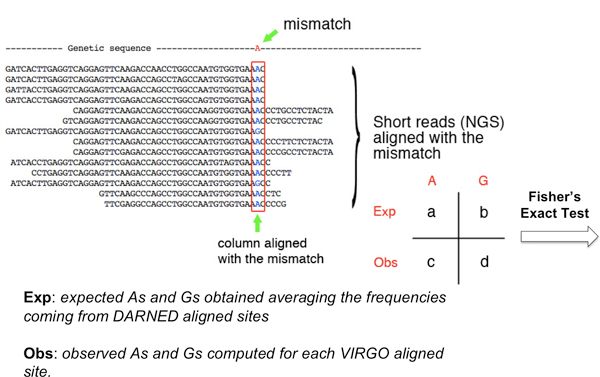
**Toy example for the p-value computation**.

Our approach to compute the p-values of candidate sites uses the expected A/G frequencies in the aligned columns versus the observed one in connection to a Fisher exact test. To compute these expected frequencies we used all the DARNED editing sites having an alignment with some NGS reads (we set to five the minimum number of reads aligning the gene region). In order to calculate the p-value, for each selected mismatch the nucleotides present in the corresponding alignment columns are considered. Only columns containing Adenosine and Guanosine are taken into account. For each editing site reported in DARNED and aligned with the NGS reads we computed the frequencies of A and G nucleotides in the column corresponding to the mismatch. Then, we take the average frequencies of A and G for all aligned DARNED editing sites. We consider as observed frequencies those coming from a mismatch visualized by VIRGO which has an alignment with NGS reads. These frequencies (expected/observed) are then used through the Fisher's Exact Test to compute the putative site p-value (see Figure 4 for an example).

The significance of those mismatches for which it was not possible to compute the p-values was annotated as unknown.

Finally, p-values have been adjusted applying FDR correction for testing multiple hypotheses, with *α *= 0.01. Each p-value is periodically updated by using new NGS experiments.

## Utility and discussion

VIRGO aims to be an efficient and user-friendly system, providing an interface by which users can analyze and visualize their data, and export results into xml and txt files.

The central purpose of *VIRGO *is to provide users with a periodically updated system which stores high-quality candidate editing sites. This will allow users to quickly and easily identify whether their genomic sequences are subject to A-to-I RNA Editing.

**Figure 5:**
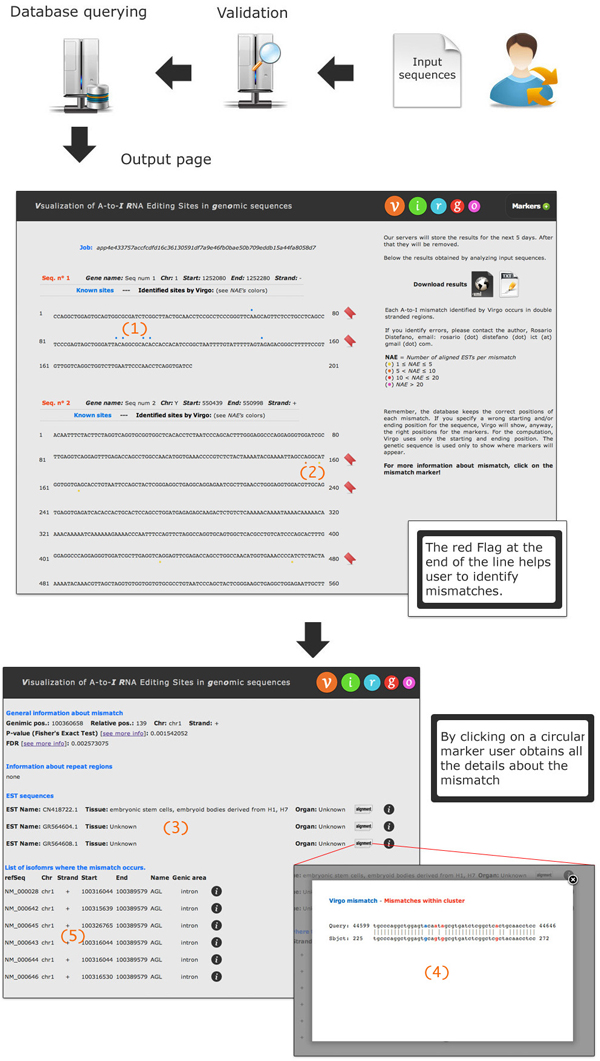
**VIRGO usage example**. Once the user provides the query sequences, VIRGO extracts mismatches stored into the knowledge base.

**Figure 6:**
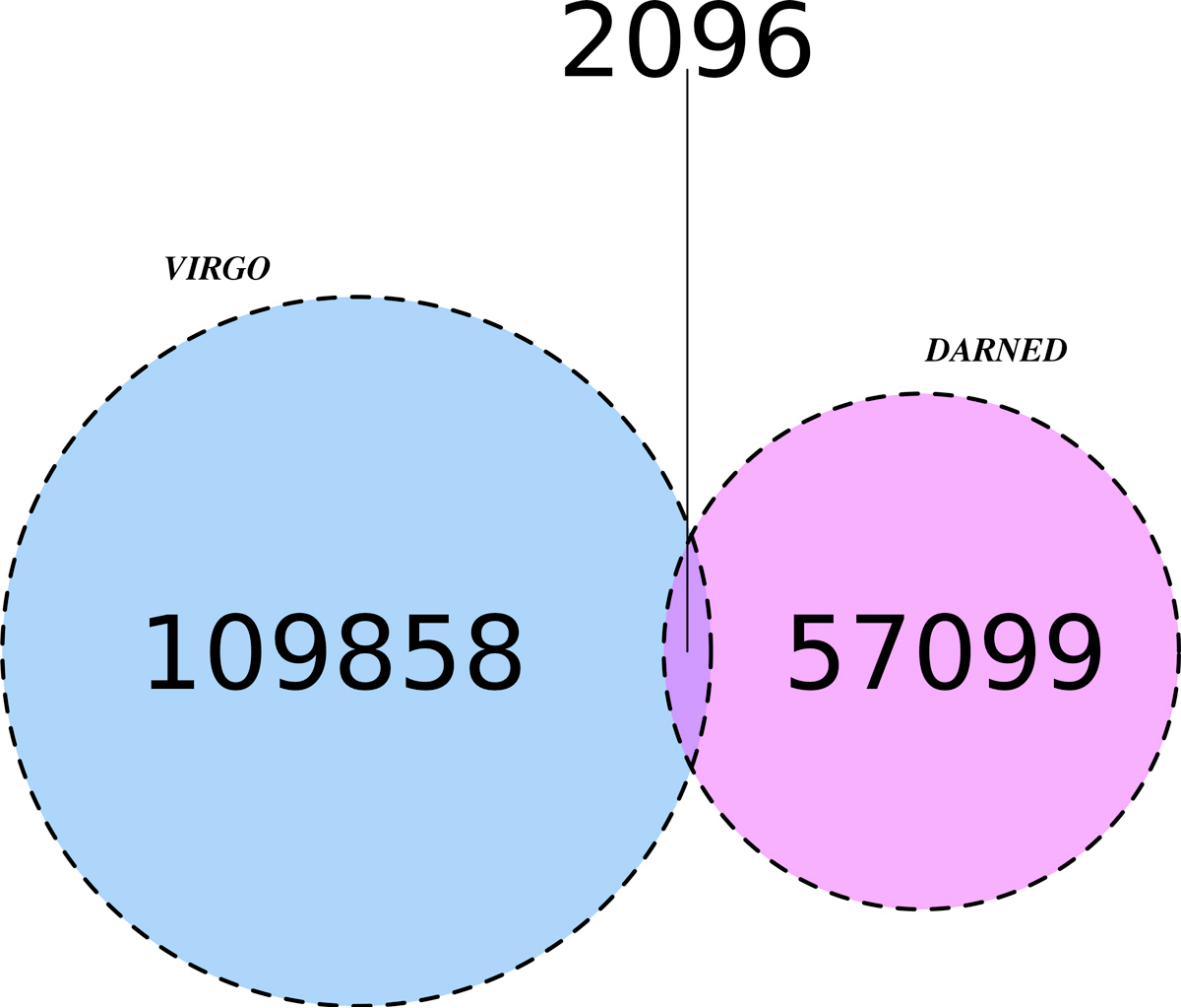
**Venn diagram concerning the number of editing sites in common between VIRGO and DARNED**.

The user can submit an input file containing headers of sequences in a specific BED-*like *format (see the website for input examples). Note that improperly formatted input sequences will not be analyzed. Once the analysis starts, a temporary page containing a link to the results page is generated (see Figure 5). The left part of the results page shows the sequences that have been analyzed. Each sequence is partitioned into segments of 80 nucleotides each. All known mismatches (obtained from DARNED) are identified by blue marks placed on top of them (see number 1 in Figure 5). In Figure 6 we show, through a Venn diagram [38], the number of common sites shared by VIRGO and DARNED. Notice that, only a small portion of VIRGO editing sites overlaps with those present in DARNED. There are several arguments to explain this. First of all, RNA-editing is a dynamic event; this means that the presence of edited adenosines can have, in principle, a strong variability. For example, a sequenced transcript can have an edited adenosine in a specific position in an experiment which is absent in the same sequenced transcript in a second experiment. This conjecture is supported by the fact that most of the data included in DARNED come from experiments in which authors synthesized their own ETSs or NGS transcripts. Within this context, tools as Virgo are useful to help investigation. A second reason relies on the fact that the second phase (clustering filter) of VIRGO hides those candidate editing events that do not happen in clusters. However, since editing is rarely an-all-or-nothing mechanism, we are confident that our dataset, being based on the actual EST sequence reads, gives an accurate measure for the editing events occurring in vivo.

The sites identified by VIRGO are marked with different colors (yellow, orange, red, purple) according to the Number of Aligned ESTs (NAEs. The colors with respect to the *NAEs *are: (*yellow*)1 ≤ *NAE *≤ 5, (*orange*)5 <*NAE *≤ 10, (*red*) 10 <*NAE *≤ 20, (*fuxia*) *NAE *≥ 20). They are placed at the bottom of sequences (see number 2 in Figure 5). By clicking on a blue marker, VIRGO shows the following information: chromosome, genomic position, strand, p-value, tissue/organ (if known), if it is a SNP and the PUBMED resources.

Markers relative to newly predicted sites will give information on chromosome, genomic position, strand, and p-value. When a mismatch occurs inside a repeat region, its start/end genomic position, strand, chromosome, name, class and family will be given. The list of EST sequences in which the mismatch occurs is given. For each EST sequence, VIRGO shows the EST name, tissue and organ (if known), the alignment between the input gene and EST sequence, and the NCBI information. The list of isoforms where the mismatch occurs is also provided. For each isoform, information such as the refSeq ID, chromosome, strand, starting and ending genomic position, among others, are provided (see number 3, 4 and 5 in Figure 5). Finally, the results of the analysis will be stored into the server for 5 days and then removed.

## Conclusions

RNA Editing is an important post-transcriptional mechanism which contributes to the diversity of transcriptome. It alters the sequence of primary RNA transcripts by deleting, inserting or modifying residues. Here we focus on A-to-I editing (Adenosine-to-Inosine), the most frequent and common one. The main goal of VIRGO is to provide a simple system aiming to identify known and putative A-to-I RNA editing sites into user provided genomic sequences. By exploiting NGS data, VIRGO is able to compute, for each predicted editing site, a p-value to measure the confidence of the prediction. Predictions can be downloaded in *xml *and *txt *format. Finally, the whole VIRGO database can be downloaded and used in third party applications.

## List of abbreviations used

NAE: Number of Aligned EST.

## Competing interests

The authors declare that they have no competing interests.

## Authors' contributions

RD, GN, and AP conceived and designed the system. RD and GN implemented the database and the web interface. VM, AL, RG, AP, and AF contributed analysis tools. RG, AP, AF supervised the project. RD, GN, AL, RG, AP and AF wrote the paper. All authors read and approved the final manuscript.

## Declarations

The publication costs for this article were funded by PO - FESR 2007-2013 grant - CUP: G23F11000840004 -Title: "BIOWINE".

This article has been published as part of *BMC Bioinformatics *Volume 14 Supplement 7, 2013: **Italian Society of Bioinformatics (BITS): Annual Meeting 2012**. The full contents of the supplement are available online at http://www.biomedcentral.com/bmcbioinformatics/supplements/14/S7
